# Human TLR8 Senses RNA From *Plasmodium falciparum*-Infected Red Blood Cells Which Is Uniquely Required for the IFN-γ Response in NK Cells

**DOI:** 10.3389/fimmu.2019.00371

**Published:** 2019-03-27

**Authors:** Christoph Coch, Benjamin Hommertgen, Thomas Zillinger, Juliane Daßler-Plenker, Bastian Putschli, Maximilian Nastaly, Beate M. Kümmerer, Johanna F. Scheunemann, Beatrix Schumak, Sabine Specht, Martin Schlee, Winfried Barchet, Achim Hoerauf, Eva Bartok, Gunther Hartmann

**Affiliations:** ^1^Department of Clinical Chemistry and Clinical Pharmacology, University Hospital Bonn, Bonn, Germany; ^2^German Center for Infection Research (DZIF), Partner Site Bonn-Cologne, Bonn, Germany; ^3^Department of Virology, University Hospital Bonn, Bonn, Germany; ^4^Institute of Medical Microbiology, Immunology and Parasitology (IMMIP), University Hospital Bonn, Bonn, Germany

**Keywords:** innate immune system, toll-like receptor 8, interleukin 12p70, interleukin 18, interferon gamma, NK cells, malaria, *Plasmodium falciparum*

## Abstract

During blood-stage malaria, the innate immune system initiates the production of pro-inflammatory cytokines, including IFN-γ, that are critical to host defense and responsible for severe disease. Nonetheless, the innate immune pathways activated during this process in human malaria remain poorly understood. Here, we identify TLR8 as an essential sensor of *Plasmodium falciparum*-infected red blood cells (iRBC). In human immune cells, iRBC and RNA purified from iRBC were detected by TLR8 but not TLR7 leading to IFN-γ induction in NK cells. While TLR7 and 9 have been shown to lead to IFN-γ in mice, our data demonstrate that TLR8 was the only TLR capable of inducing IFN-γ release in human immune cells. This unique capacity was mediated by the release of IL-12p70 and bioactive IL-18 from monocytes, the latter via a hitherto undescribed pathway. Altogether, our data are the first reported activation of TLR8 by protozoan RNA and demonstrate both the critical role of TLR8 in human blood-stage malaria and its unique functionality in the human immune system. Moreover, our study offers important evidence that mouse models alone may not be sufficient to describe the human innate immune response to malaria.

## Introduction

With 216 million clinical cases and over 400,000 deaths worldwide in 2016, malaria remains a significant global health problem (1). Of these fatalities, over two thirds occur in children under five, and 99% are due to infection with *Plasmodium falciparum* (*P. falciparum*). Severe falciparum malaria is associated with a strong pro-inflammatory response during blood-stage disease (2–5). This release of pro-inflammatory cytokines, including IL-1β, IL-12, IL-18, TNF, and IFN-γ, is seen as a “double-edged sword,” correlating with both protective immunity and disease severity (6–8). Despite important differences between murine and human malaria, murine models of malaria using rodent *Plasmodium* species recapitulate the pro-inflammatory phenotype observed in human infection (9–11) and have demonstrated a critical dependence on early IFN-γ release from natural killer (NK) cells for host defense during the blood stage (12–15). Further studies have reported that IFN-γ release during murine malaria occurs downstream of TLR7 and TLR9 signaling (16, 17) and is thus dependent on the sensing of *Plasmodium*-derived nucleic acids. Human NK cells have been observed to produce IFN-γ upon exposure to *P. falciparum*-infected red blood cells (iRBC) (18, 19), a process which is dependent on IL-12p70 and IL-18 release from accessory cells (20). In addition to NK cells, a number of reports have demonstrated that CD4+ and CD8+ αβT cells and, in particular, γδ T cells produce IFN-γ after Plasmodium infection (21–23). However, despite a broad variety of well-characterized *Plasmodium*-derived pathogen-associated molecular patterns (PAMPs) which activate known pattern recognition receptors (PRRs) (6, 24), it remains unclear which precise PAMPs and PRRs are required for IFN-γ release from human cells in the blood stage of *Plasmodium* infection.

Of note, there are important differences between murine and human innate immune sensing of nucleic acids in the endosome. While murine TLR7 and TLR9 are expressed in a broad variety of myeloid cells and their activation lead to the release of type I interferons (IFNs) and MyD88-dependent NF-κB driven cytokines, including the NF-kB/IRF1 cytokine IL-12p70, human TLR7 and TLR9 expression is largely restricted to B-cells and plasmacytoid dendritic cells (pDCs), and their activation lead to the release of type I IFNs (25–27). IL-12p70 release from human monocytes can instead be triggered by the endosomal RNA-sensor TLR8 (28, 29), which, in mice, does not function as a pro-inflammatory single-stranded RNA receptor (30, 31) and may, in fact, have an anti-inflammatory function (32, 33). Human TLR8 shares many common RNA and small-molecule ligands with TLR7, yet differential activators of TLR7 and TLR8 have been described (29, 34, 35), and recent studies utilizing CRISRPR/Cas9 genome editing in human cells have shown that human TLR8 can preferentially recognize bacterial RNA and initiate antibacterial host defense (36, 37). However, due to the lack of murine models for TLR8 function to date, we are only beginning to understand the functionality of TLR8 in the human system (38, 39).

In this study, we demonstrate that, in contrast to murine models, *P. falciparum*-derived RNA (PfRNA) but not DNA (PfDNA) is able to induce IFN-γ release from human NK cells. Moreover, our data reveal that PfRNA and *P. falciparum*-infected red blood cells (iRBC) and, in particular, PfRNA are selectively sensed by human TLR8, not TLR7, in the endolysosomal compartment, the first description of the participation of TLR8 in the sensing of eukaryotic pathogens. We provide evidence that TLR8 activation in human monocytes is essential for IFN-γ release from natural killer (NK) cells upon stimulation with PfRNA and iRBC, thus elucidating the PAMPs triggering early IFN-γ release in human malaria. Human TLR other than TLR8 were unable to induce IFN-γ in NK cells, since only iRBC, PfRNA, and other TLR8 ligands were able to both induce IL-12p70 and simultaneously prime and activate the release of bioactive IL-1β and IL-18. Thus, our study underscores the unique role of TLR8 in human host defense.

## Results

### *P. falciparum*-Infected Red Blood Cells and *P. falciparum* RNA but Not DNA Induce IFN-γ Release From Human NK Cells

**Figure 1:**
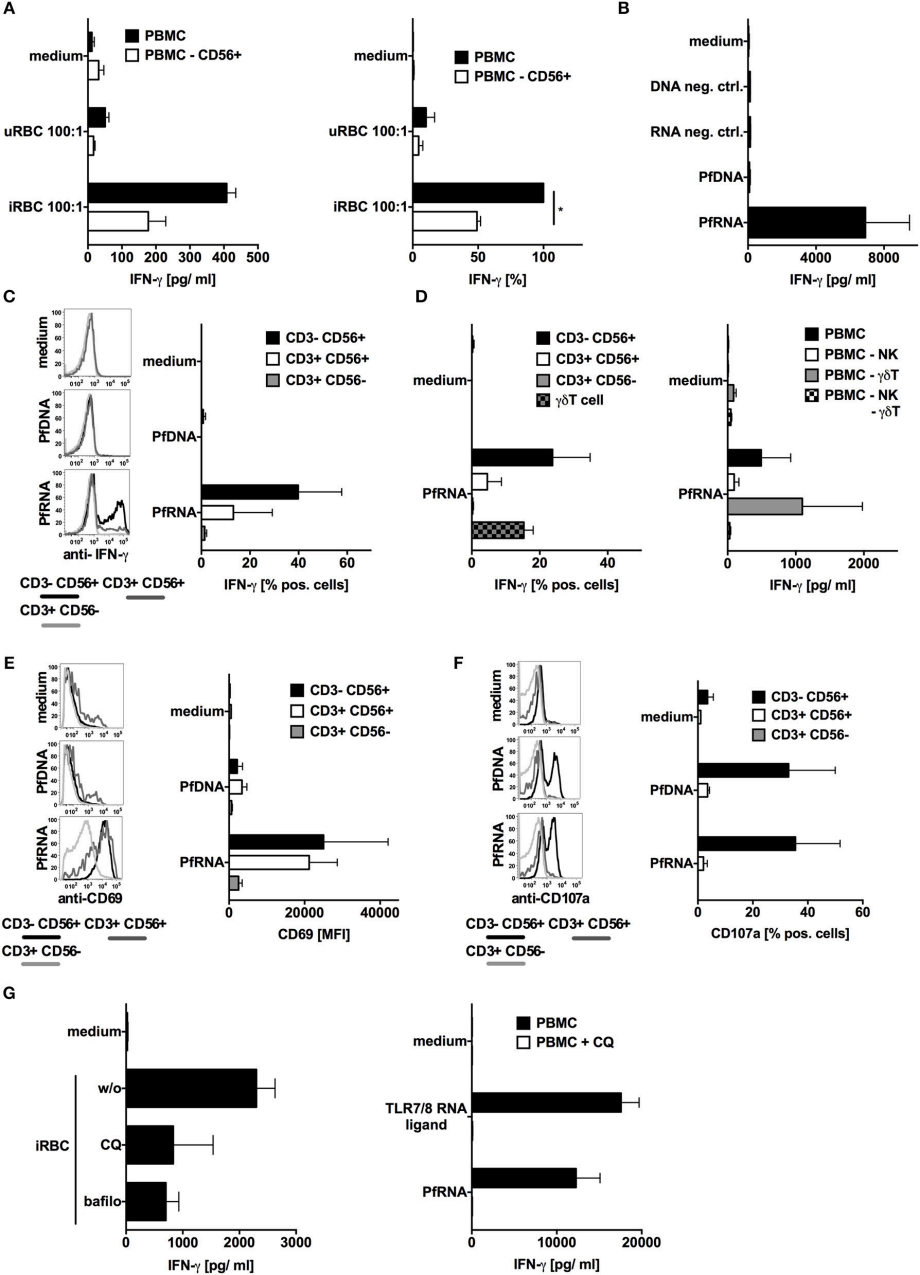
*P. falciparum*-infected red blood cells (iRBC) and *P. falciparum*-RNA (PfRNA) but not DNA (PfDNA) induce IFN-γ in human PBMC via endosomal PRRs. **(A)** Human PBMC with or w/o depletion of CD56+ cells were stimulated with iRBC or uninfected red blood cells (uRBC) as indicated. Twenty-four hours later, IFN-γ was analyzed in the supernatant. One representative donor depicted left, mean ± SEM of 5 donors in relation to iRBC on PBMC right. **(B)** Human PBMC were stimulated with PfRNA, PfDNA, or an inert RNA or DNA (neg. ctrl.). Twenty-four hours later, IFN-γ was analyzed in the supernatant. Graph shows mean ± SEM of 5 donors. **(C)** Human PBMC were stimulated with PfRNA or PfDNA, after 12 h cells were harvested, fixed, and IFN-γ expression in CD3-CD56+, CD3+CD56+, and CD3+CD56- cells was analyzed by flow cytometry. Percentage of positive cells is depicted. Graph shows mean ± SEM of *n* = 6 donors/*n* = 4 donors for PfDNA. **(D)** Done as described for **(C)** but additional γδT cells were analyzed (left graph, mean ± SEM *n* = 3) and PBMC with depleted cell subtypes as indicated were incubated for 24 h with PfDNA or PfRNA before IFN-γ was analyzed in the supernatant (right graph, ±SEM *n* = 4). **(E)** Done as described for **(C)** but cells were analyzed for surface expression of CD69 and mean fluorescence intensity is depicted. Graph shows mean ±SEM of 4 donors. **(F)** Done as described in **(C)** but after 12 h cells were blocked with Brefeldin A, incubated with 5 × 10^4^ tumor cells (A549) and analyzed by FACS for CD107a expression. Graph shows mean ± SEM of 2 donors **(G)** Human PBMC were treated with 10 μM chloroquine (CQ) or 50 nM bafilomycin (bafilo) for 1 h and then stimulated with RNA (right) or *P. falciparum*-infected (iRBC) or uninfected (uRBC) red blood cells (left) as indicated. Graph shows mean ± SEM of 4 donors. ^*^Indicates a *p* < 0.05.

TLR7 and TLR9 have been reported to contribute to innate immune sensing during blood-stage infection in murine malaria models. Whereas, *P. falciparum*-derived DNA is a known human TLR9 ligand (40, 41), the pathways involved in the sensing of *P. falciparum* RNA (PfRNA) and *P. falciparum*-infected red blood cells (iRBC) via human endosomal PRRs has not been investigated to date. To analyze this, we incubated human PBMC with or without depleted NK cells with iRBC (purity of depletion Supplementary Figure 1). As previously published (18, 19), iRBC-induced IFN-γ production in human PBMC and was substantially dependent on NK cells (Figure 1A). To determine the role of plasmodium DNA (PfDNA) and RNA (PfRNA) in this IFN-γ response and which PRRs are involved, we purified PfDNA and PfRNA from iRBC. A striking difference could be observed after direct stimulation with these iRBC-derived nucleic acids: only PfRNA but not PfDNA was able to induce IFN-γ in PBMC, thus implicating RNA but not DNA-sensing PRRs to be involved in IFN-γ release from PBMC in response to *P. falciparum* (Figure 1B and Supplementary Figure 2). As analyzed by flow cytometry, the cell subsets that are responsible for this IFN-γ release were mainly found to be NK cells and to a lesser extent NKT cells and γδT cells (Figures 1C,D). This is in line both with previous reports (18, 22) and the partial reduction of IFN-γ in PBMC in response to iRBC after depletion of NK cells seen in Figure 1A. Activation of γδT cells also requires the γδT-cell receptor, and numerous publications demonstrate the importance of the NK cell for the early immune response in the blood stage (12, 14, 18, 19). Thus, in the current manuscript, we chose to focus on the NK-cell response after exposure to plasmodial PAMPs. We additionally compared the expression of other markers of NK cell activation after stimulation with PfDNA and PfRNA. CD69 was robustly upregulated after treatment with PfRNA in NK cells but only weakly induced in response to PfDNA (Figure 1E). However, the release of cytotoxic granules was induced by both PfRNA and PfDNA in a comparable fashion (Figure 1F). Pathogenic RNA can be sensed by a number of cytosolic and endolysosomal PRRs (26). Thus, to determine whether PfRNA was sensed within the cytosolic or endosomal compartment, we treated human PBMC with chloroquine or bafilomycin before stimulating with iRBC or PfRNA. Chloroquine (CQ) and bafilomycin inhibit lysosomal acidification and thus the activation of TLRs 3, 7, 8, and 9 within the endosome of immune cells (42, 43). Both chloroquine and bafilomycin inhibited the induction of IFN-γ in response to iRBC and PfRNA (Figure 1G). A possible contamination with endotoxin could be excluded in a LAL assay (Supplementary Figure 3). Thus, our data demonstrate that IFN-γ is induced from human NK cells by PfRNA but not PfDNA, and PfRNA and iRBC are recognized by an RNA-sensing PRR within the endosomal compartment.

### *P. falciparum* RNA and *P. falciparum*-Infected Red Blood Cells Specifically Activate TLR8

**Figure 2:**
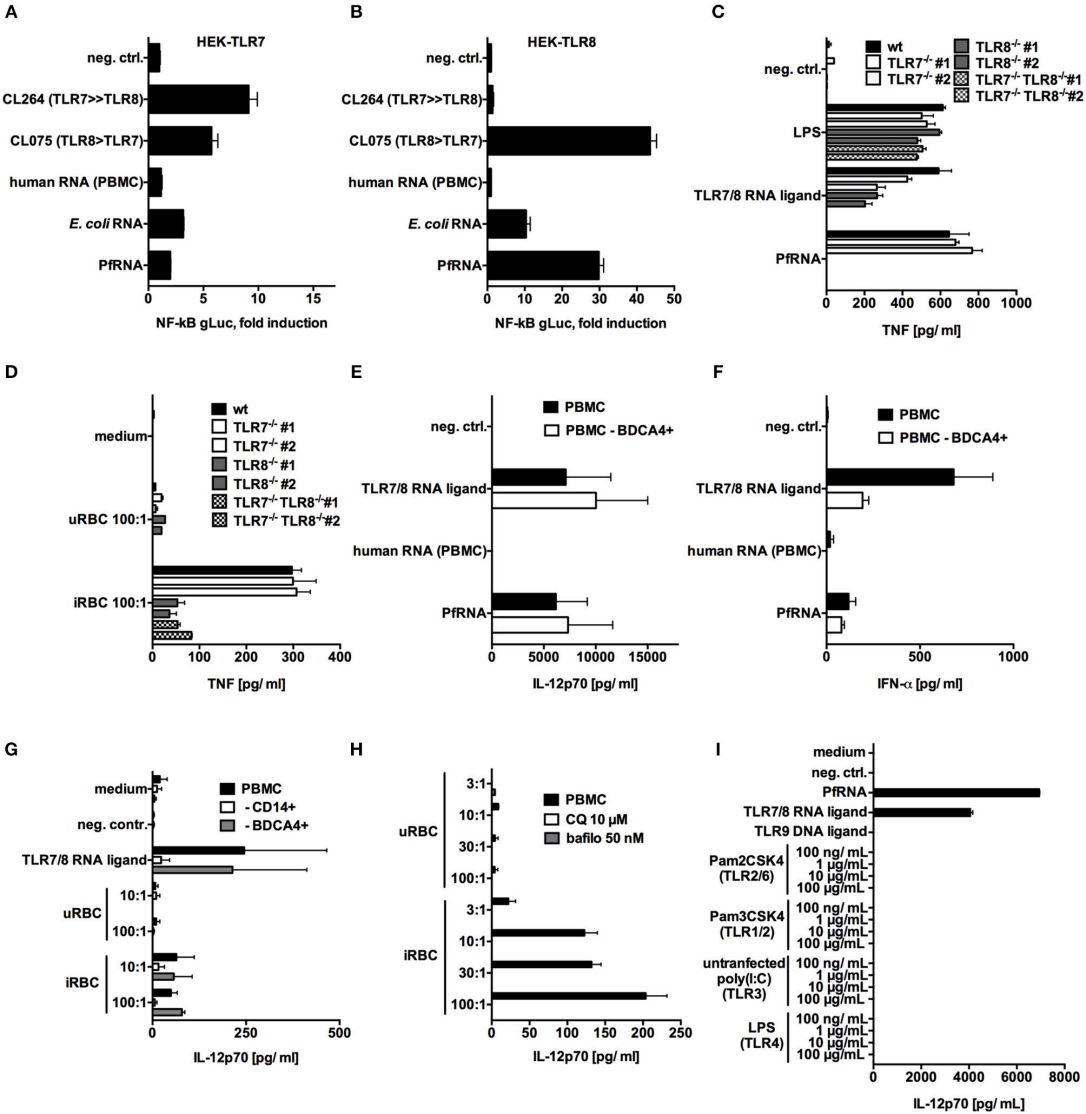
*P. falciparum* RNA (PfRNA) and *P. falciparum*-infected red blood cells specifically activate TLR8. **(A,B)** HEK 293-XL cells overexpressing TLR7 **(A)** and TLR8 **(B)** were transfected with an NF-κB-gLuc reporter plasmid and stimulated with 1 μg/mL of the small-molecule ligands CL264 and CL075 or with RNA as indicated. gLuc activity was measured in cellular supernatants after 16 h. **(C,D)** PMA-differentiated wildtype (wt) THP-1, TLR7^−/−^, TLR8 ^−/−^, or TLR7^−/−^ TLR8 ^−/−^ cells were primed with 200 U/mL IFN-γ and stimulated with RNA **(C)** or *P. falciparum*-infected (iRBC) or uninfected (uRBC) red blood cells **(D)** as indicated. Cellular supernatants were analyzed for TNF release after 20 h via ELISA. Two clonal cell lines (#1 and #2) were used for each genotype. **(E,F)** Human PBMC and PBMC depleted of cells carrying the pDC marker BDCA4 were stimulated as indicated, and cellular supernatants were analyzed after 24 h for IL-12p70 **(E)** or IFN-α **(F)** via ELISA. **(G)** Human PBMC and PBMC depleted of cells bearing the monocyte marker CD14 or the pDC marker BDCA4 were stimulated as indicated, and cellular supernatants were analyzed after 24 h for IL-12p70 via ELISA. **(H)** Human PBMC were treated with 10 μM chloroquine (CQ) or 50 nM bafilomycin (bafilo) for 1 h and then stimulated with the indicated dilutions (PBMC:RBC) of uRBC or iRBC. Cellular supernatants were analyzed for IL-12p70 after 24 h. **(I)** Human PBMC were stimulated with titrated TLR ligands indicated, and after 24 h, cellular supernatants were analyzed for IL-12p70 via ELISA. **(A–I)** Where indicated, 9.2s RNA served as a TLR7/8 ligand and poly(CA)_10_ as a negative control and CpG 2006 as TLR9 ligand. Unless otherwise indicated, iRBC and uRBC were added in a 100:1 ratio to the cells stimulated, and RNA were complexed with p-L-Arginine before transfection. Data shown are mean ± SD and representative of 3 experiments **(A–D)** or mean ± SEM of 2 **(G)** or 4 **(E,F,H,I)** compiled donors.

The single-stranded RNA(ssRNA) sensor TLR7 has been reported to act as an early sensor of plasmodium infection (17), and TLR7-deficient mice are partially protected from cerebral malaria (44). In human cells, ssRNA in the endosome can be sensed by both TLR7 and TLR8. Thus, we attempted to determine the respective involvement of these two PRRs in PfRNA and iRBC sensing. To specifically analyze the interaction of PfRNA with TLR7 and TLR8, we utilized HEK293-XL cell lines (Invivogen) overexpressing human TLR7 or TLR8 (HEK-TLR7, HEK-TLR8). PfRNA, human PBMC total RNA and *E. coli* RNA were respectively co-transfected with a gaussia luciferase (gLuc)-based NF-kB reporter into HEK-TLR7 and HEK-TLR8 (Figures 2A,B). The small-molecule agonists of TLR7 and 8 (CL075-TLR7/8; CL264-TLR7) and the inert RNA poly(CA)_10_ (45) were used as controls. As expected, a robust gLuc signal in both HEK-TLR7 and HEK-TLR8 cells was observed for CL075; CL264 induced a stronger signal in HEK-TLR7, and human total RNA and poly(CA)_10_ (neg. ctrl.) showed only limited activity. In contrast, PfRNA induced a much stronger gLuc signal in HEK-TLR8 cells (Figure 2B) than in HEK-TLR7 (Figure 2A), indicative of a selective ability to activate TLR8. Strikingly, this differential activation of TLR8 was even more pronounced than that of *E. coli* total RNA, which has been already described as a preferential ligand of TLR8 (37).

Next, to examine TLR7 and TLR8 activation under endogenous, endosomal conditions, we also generated TLR7-deficient (TLR7^−/−^), TLR8-deficient (TLR8^−/−^), and double-deficient (TLR7^−/−^TLR8^−/−^) monocytic THP-1 cell lines using CRISPR/Cas9 genome editing technology (46) (Supplementary Table 1). For experiments with purified PfRNA and iRBC, cells were then subjected to stimulation with IFN-γ and PMA in order to upregulate TLR7 expression, and, in this setting, both TLR7 and TLR8 activation lead to TNF release (47). The dual TLR7/8 ligand 9.2s RNA (48) was used as a positive control and induced TNF release in WT, TLR7^−/−^, TLR8^−/−^ but not TLR7^−/−^TLR8^−/−^ THP-1 cells. In contrast, endosomal delivery of PfRNA only induced TNF in wildtype and TLR7-deficient cells, but not in TLR8^−/−^ or TLR7^−/−^TLR8^−/−^ cell lines (Figure 2C). Thus, in line with the differential activation of TLR8 by PfRNA seen in the HEK reporter cell lines, TNF release from THP-1 cells after PfRNA stimulation critically depended on TLR8.

Since PMA-differentiated THP-1 are highly phagocytic (49), we also used the TLR-reporter cell lines to investigate the innate immune response and sensing of PfRNA upon uptake of intact iRBC. We incubated primed THP-1 cells with iRBC, and, as seen with PfRNA, TNF levels were significantly reduced in TLR8^−/−^ and TLR7^−/−^TLR8^−/−^ but not TLR7^−/−^ deficient cells (Figure 2D), thus demonstrating that iRBC are capable of TLR8 but not TLR7 activation. Of note, TNF release was not completely abrogated in TLR8-deficient lines, which may be due to TNF release downstream of TLR2 or TLR4 signaling, as has been described for glycosylphosphatidylinositol (GPI) anchors in *P. falciparum* (6).

Next, we investigated whether we could recapitulate these findings in PBMC and primary human monocytes. To this end, human PBMC were stimulated via endosomal delivery of PfRNA with 9.2s RNA as a TLR7/8 positive control. TLR8 stimulation is known to induce high levels of TNF and IL-12p70 release, while TLR7 activation in human PBMC induces high levels of IFN-α (27). As expected, endosomal delivered 9.2s RNA induced robust activation of TNF (Supplementary Figure 2A) and IL-12p70 (Figure 2E), indicative of TLR8 activation, and IFN- α, indicative of TLR7 signaling (Figure 2F). In contrast, while PfRNA stimulation induced TNF (Supplementary Figure 4A) and IL-12p70 (Figure 1E), it only resulted in limited induction of IFN-α (Figure 2F). Moreover, IFN-α release, but not IL-12p70, was reduced after pDC depletion from PBMC (Figures 2E,F and Supplementary Figure 1), which is in line with previous studies reporting robust expression of TLR7 on pDC (50, 51). Since human monocytes express high levels of TLR8, CD14+ cells were purified from PBMC and stimulated with 9.2s RNA and PfRNA. As expected, primary monocytes demonstrated robust IL-12p70 induction upon stimulation with both RNA ligands (Supplementary Figure 4B).

We then examined whether stimulation of PBMC with intact iRBC resulted in a pattern similar to what was observed for purified PfRNA. We incubated PBMC with iRBC, which led to IL-12p70 induction, in line with previously published data (20) (Figure 2G). In addition, we could also confirm previously published data in which depletion of monocytes, but not of pDCs, reduced levels of IL-12p70 release upon exposure to iRBC (Figure 2G and Supplementary Figure 1). In contrast to endosomal delivery of defined nucleic acids, whole pathogen exposure can potentially activate a variety of PRRs. In particular, *Plasmodium* RNA from sporozoites is known to activate MDA5 in the cytosol of murine hepatocytes during the liver stage of *Plasmodium* infection (52). Thus, we investigated whether iRBC-induced IL12p70 release was indeed dependent on endosomal sensing. To this end, we used bafilomycin and chloroquine, known inhibitors of endosomal acidification and endosomal TLR signaling (42, 43). Application of bafilomycin and chloroquine abrogated iRBC-induced IL-12p70 (Figure 2H), indicating that an endosomal receptor is critically required for IL-12p70 release in response to the sensing of iRBC in human PBMC. Moreover, we investigated the ability of other TLR agonists to induce IL-12p70. Strikingly, only the stimuli with TLR8 agonist activity, 9.2s RNA (TLR7/8) and PfRNA (TLR8), but not other TLRs (TLR1/2, TLR2/6, TLR3, TLR4, TLR9), induced robust IL-12p70 activation (Figure 2I), providing a strong indication that other PAMPs in iRBC, such as DNA (TLR9 ligand) or GPI (TLR2/4 agonist), are not able to contribute to iRBC-induced IL-12p70 release via TLR activation in human PBMC. In line with this, DNA derived from iRBC was unable to induce IL-12p70 (Supplementary Figure 4C). Altogether, these data indicate that PfRNA specifically activates TLR8 and that iRBC are sensed via *Plasmodium* RNA, leading to TLR8 activation and IL-12p70 release in human PBMC.

### *Plasmodium* RNA and Infected Red Blood Cells Induce the Release of Bioactive IL-1β and IL-18 via TLR8

In addition to IL-12p70, the inflammasome cytokines IL-1β and IL-18 have often been implicated in NK cell activation and IFN-γ release, with bioactive IL-18 originally known as “IFN-γ inducing factor” (53–56). It has also been previously reported that bioactive IL-18 is released from PBMC after exposure to iRBC and participates in IFN-γ induction (20). Thus, we wanted to investigate whether IL-1β and IL-18 release from PBMC after exposure to PfRNA and iRBC were also dependent on TLR8. Moreover, since inflammasome components can also be upregulated or “primed” via TLR stimulation before their posttranslational activation (57, 58), we also specifically examined whether TLR8 only contributed to the initial step of inflammasome priming or also directly to the activation of IL-1β and IL-18, as it has been previously described for TLR4 activation in monocytes (59).

**Figure 3:**
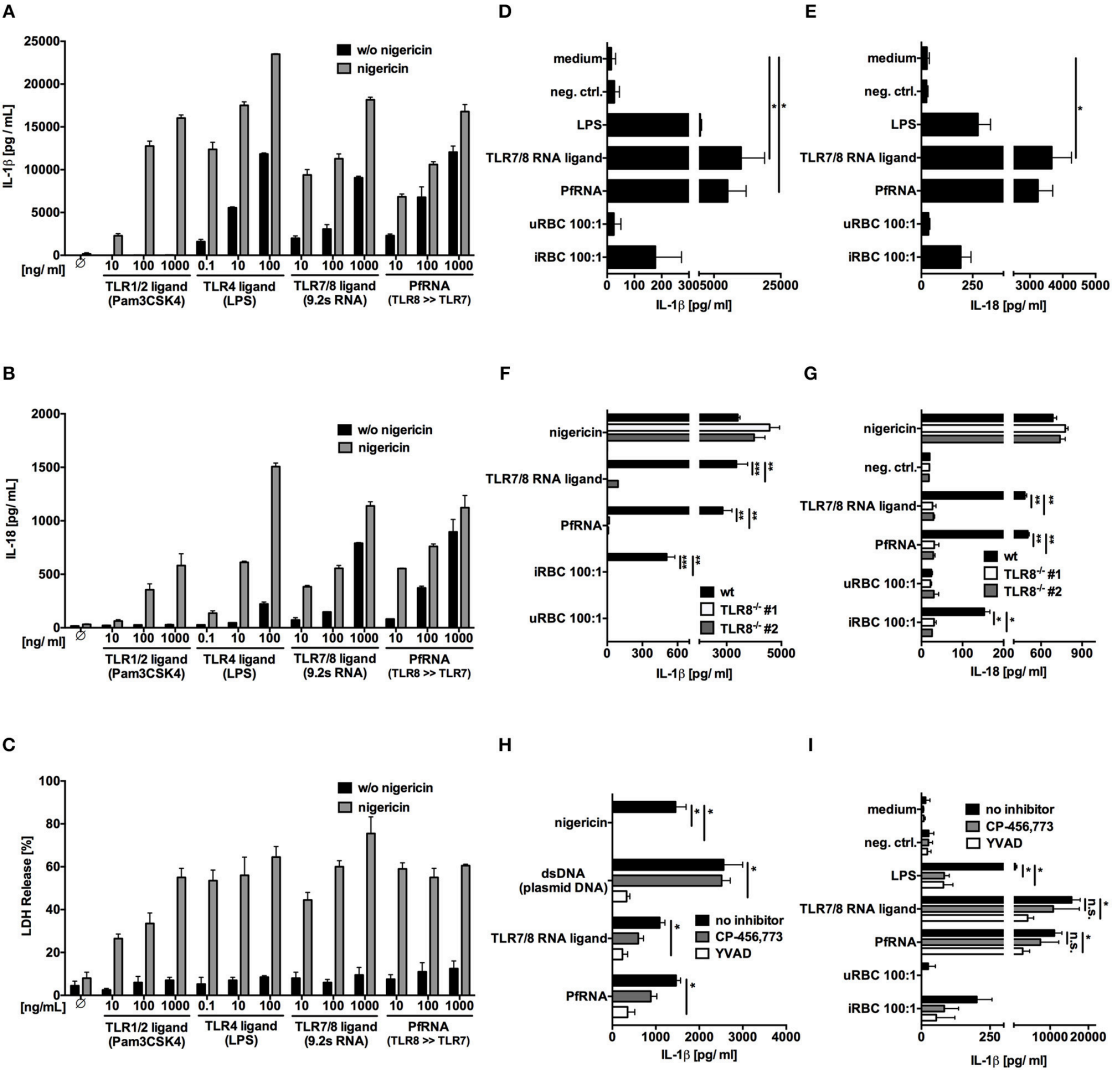
*Plasmodium* RNA (PfRNA) and infected red blood (iRBC) induce the release of bioactive IL-1β and IL-18 via TLR8. **(A–C)** Human PBMC were stimulated with TLR ligands and PfRNA as indicated. After 4 h (LPS, Pam3Cys) or 8 h (RNA ligands), cells were stimulated with 5 μM nigericin, an NLRP3 activator. After 12 h, cellular supernatants were analyzed for IL-1β **(A)** and IL-18 **(B)** release via ELISA and lactate dehydrogenase (LDH) activity **(C)**. **(D,E)** Purified human monocytes were stimulated as indicated. After 16 h, cellular supernatants were analyzed for IL-1β **(D)** and IL-18 **(E)** release via ELISA. **(F,G)** PMA-differentiated wildtype (wt) and TLR8^−/−^ THP-1 were stimulated as indicated. After 16 h, cellular supernatants were analyzed for IL-1β **(F)** and IL-18 **(G)** release via ELISA. Two clonal cell lines (#1 and #2) were used for each genotype. **(H,I)** PMA-differentiated THP-1**(H)** and purified human monocytes **(I)** were incubated with the NLRP3 inhibitor CP-456,773 (5μM) or the Caspase-1 inhibitor YVAD (20μM) for 1 h prior to stimulation. After 16 h, cellular supernatants were analyzed for IL-1β via ELISA. **(A–I)** Where indicated, 9.2s RNA served as a TLR7/8 ligand and poly(CA)_10_ as a negative control. Unless otherwise indicated, iRBC and uRBC were added in a 100:1 ratio to the cells stimulated, and RNA were complexed with p-L-Arginine before transfection. Data shown are mean ± SEM of 4 **(A–E,I)** compiled donors or mean ± SD and representative of 3 independent experiments **(F–H)**. ^*^Indicates a *p* < 0.05, ^**^p < 0.01, and ^***^p < 0.001.

To this end, we isolated human PBMC and incubated them with increasing concentrations of Pam3Cys (TLR1/2 agonist), LPS (TLR4 agonist), 9.2s RNA (TLR7/8 agonist), and PfRNA (Figures 3A–C). After priming, cells were stimulated with the potassium ionophore and NLRP3-inflammasome activator nigericin (60, 61). Classical inflammasome priming leads to upregulation of proIL-1β, NLRP3 and other inflammasome proteins but not to IL-1β or IL-18 release (57), which is dependent on their proteolytic activation and non-canonical secretion. While all TLR agonists could prime IL-1β and IL-18 for release after nigericin stimulation, only the TLR4 agonist LPS and the TLR8 agonists 9.2s RNA and PfRNA could directly induce the release of IL-1β (Figure 3A) and IL-18 (Figure 3B) on their own without addition of nigericin, suggesting that activation of TLR8, like TLR4, could not only prime but also activate the inflammasome. Strikingly, as observed with the TLR4-dependent “alternative inflammasome,” TLR8 activation alone did not induce pyroptotic cell death, as demonstrated by a lack of lactate dehydrogenase (LDH) release from stimulated cells (62) (Figure 3C). To rule out secondary effects, we isolated primary monocytes and stimulated them with PfRNA, 9.2s RNA, LPS, and iRBC without prior LPS priming, recapitulating what we had observed in complete PBMC and demonstrating that iRBC are capable of direct inflammasome activation (Figures 3D,E).

To determine whether this process was indeed dependent on TLR8 itself, PMA-differentiated wildtype and TLR8^−/−^ THP-1 cells were stimulated with 9.2s RNA, PfRNA and iRBC. The inert RNA poly(CA)_10_ and uninfected red blood cells (uRBC) were used as negative control and nigericin as a TLR8-independent NLRP3 stimulus. Once again, 9.2s RNA and PfRNA could directly induce release of IL-1β (Figure 3F) and IL-18 (Figure 3G), which, strikingly was abrogated in the absence of TLR8. Of note, although it has been reported that the TLR4-dependent alternative inflammasome is not active in THP-1 cells (59), our data demonstrates TLR8-mediated IL-1β and IL-18 release is functional in this cell line.

The alternative TLR4 inflammasome pathway ultimately leads to NLRP3 and caspase-1 activation (59). To investigate a possible role for NLRP3 or caspase-1 in TLR8-mediated inflammasome activation, we used the specific NLRP3 inhibitor (CP-456,773) (63) and the caspase-1 inhibitor YVAD. In THP-1 cells, AIM2 activation via dsDNA served as a NLRP3 independent control (64, 65) and NLRP3 activation via nigericin as a positive control for CP-456,773 activity. Here, like AIM2, TLR8-dependent IL-1β release demonstrated a partial dependence on caspase-1 (Figure 3H), but unlike AIM2, a partial dependence on NLRP3 as well. Surprisingly, NLRP3 and caspase-1 inhibition was even less effective in PBMC (data not shown) and purified monocytes (Figure 3I), strongly suggesting that TLR8 activates a hitherto uncharacterized, non-canonical, NLRP3-independent pathway leading to both IL-1β and IL-18 release.

### TLR8 Activation Is Necessary and Sufficient for IL-12p70 and IL-18 Release and IFN-γ Induction in Response to *P. falciparum*-Infected Red Blood Cells

Our data demonstrate that PfRNA and iRBC induce IFN-γ release from human NK cells downstream from an endosomal PRR (Figure 1) and that TLR8 induces IL-12p70, IL-1β, and IL-18 in human monocytes (Figures 2, 3). As it has been previously reported that iRBC can induce IFN-γ from NK cells in a process requiring IL-12p70 and IL-18 (18, 19), we investigated whether TLR8 is the receptor responsible for the release of IFN-γ from human NK cells.

**Figure 4:**
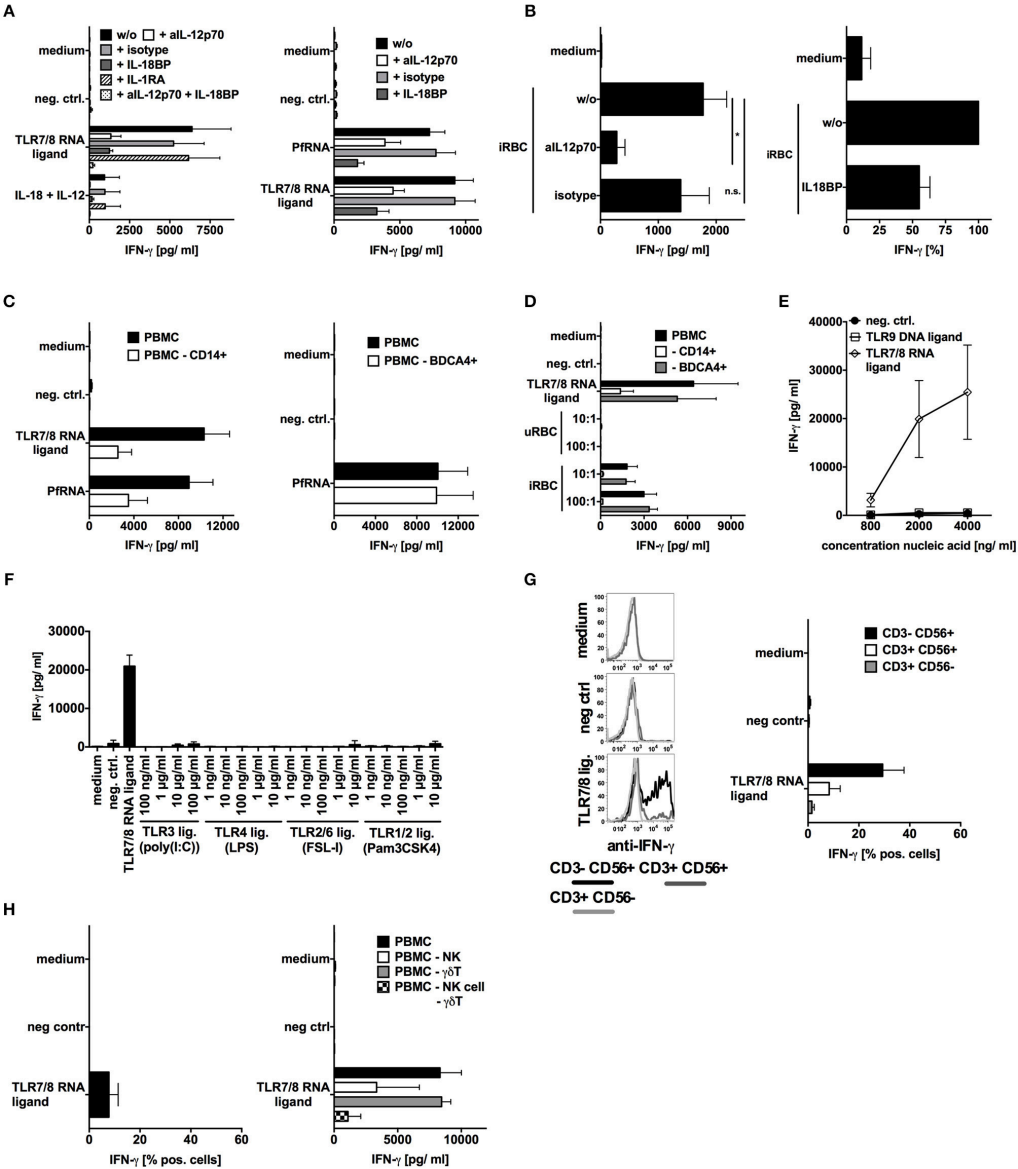
TLR8 activation is necessary and sufficient for IL-12p70 and IL-18 release and IFN-γ induction in response to falciparum should be replaced with *p. falciparum*-infected red blood cells (iRBC). **(A,B)** Human PBMC were treated with inhibitors of IL-12p70 (500ng/mL), IL-1β(10μg/mL) or IL-18BP (100ng/mL) signaling as indicated and then stimulated with TLR ligands and PfRNA as indicated. Addition of recombinant IL-12p70 and recombinant IL-18 served as positive control. **(C,D)** Human PBMC with or w/o depletion of either CD14+ or BDCA4+ cells were stimulated as indicated. **(E,F)** Human PBMC were stimulated with different amounts of TLR ligands as indicated. **(A–E)** Unless otherwise indicated, iRBC and uRBC were added in a 100:1 ratio to the cells stimulated, and RNA ligands were complexed with p-L-Arginine. Where indicated, 9.2s RNA served as a TLR7/8 ligand and poly(CA)_10_ as a negative control and CpG 2006 as TLR9 ligand. All cellular supernatants were harvested after 24 h and analyzed for IFN-γ release. The data shown are mean ± SEM of 4 compiled donors. **(G)** Human PBMC were stimulated with the TLR7/8 ligand 9.2s RNA or poly(CA)_10_ as negative control. After 12 h cells were harvested, fixed, and IFN-γ expression in CD3-CD56+, CD3+CD56+, and CD3+CD56- cells was analyzed by flow cytometry. Percentage of positive cells is depicted. Graph shows mean ± SEM of *n* = 6 donors. **(H)** Left graph done as described in **(G)** but γδT cells are shown (mean ± SEM of 3 compiled donors), right graph shows IFN-γ release in by PBMC with or without depleted NK cells or γδT cells or both after stimulation with the TLR7/8 ligand 9.2s RNA. ^*^Indicates a *p* < 0.05.

For this, we then analyzed the role of IL-12p70, IL-1β, and IL-18 by blocking their respective signaling pathways using an anti-IL-12 antibody, IL-1 receptor antagonist (IL-1RA) and IL-18 binding protein (IL-18BP) (Figures 4A,B). In concurrence with previously published studies (20, 53), IL-12p70 and IL-18 were both critical factors for IFN-γ induction by PfRNA as well as by iRBC. Moreover, the depletion of monocytes, which produce IL-12p70 and IL-18 upon TLR8 activation, significantly reduced IFN-γ induction whereas depletion of pDCs had no effect (Figures 4C,D and Supplementary Figure 1).

To elucidate a possible role for other iRBC-derived PAMPs in IFN-γ induction, we tested a broad variety of specific TLR ligands (as in Figure 2I) as well as DNA isolated from iRBC (potential TLR9 ligand) for their ability to induce IFN-γ in PBMC. Only TLR8 agonists could induce IFN-γ (Figures 4E,F). This is completely in line with our observations that, whereas robust IL-18 production is achieved by TLR4 and TLR8, only TLR8 can robustly induce IL-12p70. Moreover, it supports our previous observation that solely PfRNA but not PfDNA can induce IFN-γ in human NK cells (as in Figures 1B,C). Again, as also shown for PfRNA, synthetic RNA activating TLR7/8 induced the strongest response in NK cells but also some IFN-γ release from γδT cells and NKT cells (Figures 4G,H). Although our data indicate that TLR8 is critically required for IFN-γ release by NK cells after stimulation with PfRNA, other receptors and cytokines could still possibly modulate this process. Our data also demonstrate, PfRNA can weakly activate TLR7 and induce type I IFN (Figure 2F). To characterize the contribution of type-I IFN to type II (IFN-γ) release, we blocked type I IFN signaling using the physiological inhibitor B18R and then stimulated PBMC with a TLR7/8 agonist and PfRNA (Supplementary Figures 5A,B). In concentrations of B18R sufficient to abrogate type I IFN recognition by ELISA, there was a small reduction in IFN-γ by B18R in response to the TLR7/8 ligand and PfRNA. This seems to indicate TLR8 induced IFN-γ from NK cells can be boosted by type-I IFN, particularly during simultaneous TLR7 and TLR8 activation. Of note, a broad variety of TLR agonists could not induce IFN-γ in NK cells but could still upregulate activation markers on the surface of NK cells (CD69) and degranulation (CD107a) of NK cells (Supplementary Figures 6A–E), in agreement with other publications. However, while TLR9 agonists are capable of inducing IFN-γ in murine splenocytes (Supplementary Figure 6F), they do not induce IFN-γ in human PBMC (Figure 4E). Thus, our data highlight an important difference in the pathways to IFN-γ induction in mice and humans as well as the unique role of TLR8 in human pathogen sensing.

## Discussion

The innate immune system must discriminate between foreign and host molecules in order to effectively recognize pathogenic threats. Ideally, this immune recognition should lead to a host response tailored to the elimination of the specific pathogen. IFN-γ, also known as “macrophage-activating factor,” induces a broad range of bactericidal effects and cytotoxic immunity (66, 67). In the context of malaria, but also during infection with intracellular bacteria, IFN-γ facilitates the phagocytosis and elimination of microbes or infected cells, as well as the opsonisation and internalization of iRBC and extracellular pathogens, limiting the spread of infection (7). Recent studies have suggested that TLR8 is critical for the sensing of bacterial RNA (36, 37) and the host defense against several strains of intracellular bacteria (68–70). Our study broadens this repertoire by demonstrating that TLR8 can also detect protozoan RNA, as demonstrated for the intracellular eukaryotic protozoan *P. falciparum*. These data thus underscore the relevance of TLR8 for host defense against intracellular pathogens via IFN-γ induction.

Interestingly, in cultured human immune cells, TLR8 activation but none of the other tested TLR was able to induce IFN-γ in NK cells. This singular connection between TLR8 activation and IFN-γ is the result of the unique ability of TLR8 to induce two key IFN-γ inducing cytokines in human monocytes, IL-12p70 and IL-18, and the induction of these two cytokines represents two particularities of TLR8 signaling. Our results demonstrate that TLR8 is the only human TLR capable of inducing IL-12p70 (Figure 2I). IL-12p70 is a dimeric cytokine, consisting of two subunits: IL-12p40, which is regulated by NF-kB and can be transcriptionally activated by many TLRs, including TLR4 and IL-12p35, which is reportedly regulated by IRF1 (71). Little is known about IRF1 activation downstream of TLR8 signaling. Adding to this ambiguity is the fact that many previous papers report LPS-induced production of bioactive IL-12p70, which we could not reproduce when using ultrapure LPS (Figure 2I), despite its ability to induce IL-12p40 (data not shown). These differences may be attributable to improvements in LPS purification protocols. Thus, it is will be necessary to use well-characterized ligands when elucidating TLR-specific signaling pathways such as the one leading to the production and release of IL-12p70. It should also be noted that, in our study, we used freshly prepared, intact iRBC to investigate TLR8 activation. In contrast, erythrocyte lysis is necessary for the release of GPI anchors, which activate TLR1/2, TLR2/6 and TLR4. Thus, the storage and treatment of iRBCs may affect which cytokines are produced by iRBC cultures.

The second distinctive feature of TLR8 contributing to IFN-γ-induction is the ability of TLR8 to both prime and activate IL-18 release. Although alternative inflammasome activation has been reported for TLR4 (59), and several publications have described the concerted activation of TLR8 and NLRP3 with distinct steps for priming and activation (72), our study is the first to describe an alternative pathway of direct inflammasome activation by TLR8. Of note, TLR4-dependent alternative inflammasome activation is absent in mice, and murine TLR8 does not induce pro-inflammatory cytokines upon exposure to ssRNA. Thus, the TLR8-IL-1/IL-18 axis contributes yet another difference to the immune sensing between mice and humans, and specifically to the immune sensing of *Plasmodium* parasites in murine and human hosts, which is highly relevant for the development of future models of malaria in mice, and for further elucidating the immunopathogenesis of malaria in humans.

Another species-specific difference is the response to TLR9. In contrast to murine TLR9, human TLR9 is unable to induce IFN-γ in NK cells in response to a specific ligand or to PfDNA. However, PfDNA does potently induce other cytokines, including type I IFNs. Moreover, a recent study has demonstrated that DNA from extracellular vesicles released by iRBC can also activate the STING/IRF3 pathway leading to type I IFNs release (73). Thus, while PfDNA does not induce IFN-γ, it may still act as an important PAMP during human malaria. Indeed, type I IFNs have been identified as a pivotal immunoregulator of blood-stage malaria (74, 75). In contrast to IFN-γ, type I IFNs were found to suppress innate and adaptive responses in controlled human malaria studies, inducing the anti-inflammatory cytokine IL-10 and limiting IFN-γ release (76). Thus, it will be important to elucidate the precise contribution of STING, TLR9 and type I IFNs to immune sensing of *Plasmodium* parasites in humans and how these pathways act in concert with TLR8 to promote host defense and determine disease severity.

As with all mechanisms of host defense, excessive or chronic activation of innate immune pathways can cause immunopathology and disease. This is particularly true for malaria tropica resulting from *P. falciparum* infection, where the extent of the immune response significantly contributes to the severity and lethality of the disease. In this study, we have identified and characterized a central mechanism by which the malaria parasite *P. falciparum* activates the innate immune system of the host, providing new approaches to controlling and modulating *P. falciparum*-induced inflammation, with the goal of preserving immune defense yet mitigating damage to the host. Here, future studies on the mechanisms of TLR8 signaling may prove crucial to the development of new therapies for malaria and bacterial infections. Moreover, our results highlight the species dependence of the immune response to *Plasmodium* parasites during malaria and the importance of using human immune models in malaria research.

## Materials and Methods

### DNA-, RNA-Oligonucleotides, and TLR Ligands

DNA-oligos CpG2006 (5′-tcgtcgttttgtcgttttgtcgtt; phosphorothioate-linkages) and negative control C20-DNA (5′-cccccccccccccccccccc) were purchased from Metabion (Martinsried, Germany). The following RNA-oligos were used: 9.2s RNA (TLR7/8 ligand 5′-AGCUUAACCUGUCCUUCAA) (48), negative control A20-RNA (5′-AAAAAAAAAAAAAAAAAAAA) [all Biomers (Ulm, Germany)]; *E. coli* LPS was obtained from Sigma-Aldrich (Schnelldorf, Germany), nigericin, polyI:C, ultrapure LPS, Pam2Cys, Pam3Cys, FSL-I, CL075 and CL264 were from InvivoGen (Toulouse, France).

### Preparation, Isolation, and Culture of Cells

PBMC were prepared from buffy coats by density gradient centrifugation using Ficoll separating solution (Biochrom, Cambridge, U.K.), and lysis of RBCs was performed using BD Pharm Lyse (BD Pharmingen). Cell viability exceeded 95% as examined by trypan blue staining. 4 × 10^5^ PBMC were cultured in 200 μl RPMI 1640 medium (Biochrom, Berlin, Germany) 10% (v/v) FCS (Thermo Fisher Scientific, Oberhausen, Germany), 1 mM L-glutamine, 100 U/ml penicillin and 100 μg/ml streptomycin (Sigma-Aldrich, Schnelldorf, Germany) in 96-well plates. CD14^+^ monocytes, BDCA-4^+^ plasmacytoid dendritic cells and NK cells were depleted from PBMC using CD14 and BDCA4-microbeads and NK cell isolation Kit and monocytes were isolated using CD14 microbeads according to the manufacturer's recommendations (all Miltenyi Biotec, Bergisch Gladbach, Germany). Flow cytometry analysis revealed a purity of > 95% for isolated monocytes and a depletion of >90% of γδ T cells or > 95% of NK+ or CD14+ cells (Supplementary Figures 1A-C). As already a low number of pDC are already known to release substantial amounts of cytokines, depletion of pDC was also functionally tested via stimulation with a TLR9 ligand followed by measurement of IFN-α release (Supplementary Figure 1D). After pDC depletion IFN-α induction was completely lost. THP-1 cells were propagated in RPMI 1640 medium supplemented with 10% FBS, 1 mM sodium pyruvate, 1 mM non-essential amino acids, 100 U/ml penicillin and 100 μg/ml streptomycin (complete RPMI). For TLR7/8 stimulation, THP-1 cells were differentiated into macrophage-like cells by treatment with 300 ng/ml PMA (Sigma-Aldrich, Schnelldorf, Germany) for 4 h, washed three times with PBS and plated at 8 × 10^4^ cells per well in 96-well plates (200 μl volume per well). Forty-eight hours later, cells were washed twice with prewarmed complete RPMI 1640 medium and treated with 200 U/ml IFN-γ for 6h, followed by two additional medium changes and stimulation [adapted from (47)]. TLR7 and TLR8-expressing HEK293-XL cell lines (InvivoGen, Toulouse, France) were cultured according to the manufacturer's protocol.

### Gene-Editing of THP-1 Cells

THP-1 cells were electroporated with plasmids expressing EF1-alpha promoter driven Cas9-NLS-2A-EGFP and U6-guideRNAs targeting TLR7 [GATGTCTGGTATGTGGTTAA(TGG)] and TLR8 [GTGCAGCAATCGTCGACTAC(AGG)], respectively, using the Neon Transfection System (Thermo Fisher Scientific, Darmstadt, Germany) at 1,250 V, 50 ms, 1 pulse. Cells were sorted for EGFP expression and single-cell clones were validated by Sanger sequencing of PCR-amplified genomic loci and functional testing with TLR7/8 specific small molecules (Invivogen, Toulouse, France). TLR7/8 double KOs were generated by consecutive targeting of TLR7 and TLR8 (46).

### Culture and Isolation of *Plasmodium* Parasites

The 3D7 strain of *P. falciparum* was cultured in continuous culture in petri dishes at 37°C with a gaseous phase of 90% N2, 5% O_2_, and 5% CO_2_, according to (77). Parasites were cultured in fresh human red blood cells from A+ donors in RPMI 1640 medium (Sigma-Aldrich, Schnelldorf, Germany) supplemented with 25 mM HEPES, 20 mM sodium bicarbonate, and 10% heat-inactivated human A+ plasma at 10% (v/v) hematocrit. The parasitemia of the infected red blood cells (iRBC) was determined by light microscopy and estimated by Giemsa-stained smears as previously described. Non-synchronized parasite cultures reaching 4–6% total parasitized erythrocytes were used that contained all parasite stages including schizonts (2.5–4% of all iRBC). The cultures were tested by PCR and found to be free of mycoplasma. Freshly prepared iRBC were used for all stimulation experiments without interim storage or freezing.

### Purification of Nucleic Acids

Plasmodium cultures with a parasitemia of >5% were used for nucleic acid isolation. Total RNA from *E. coli* DH10B and human PBMC was isolated with Trizol Reagent (Thermo Fisher Scientific, Oberhausen, Germany) and subjected to digestion with DNAse (Thermo Fisher Scientific, Oberhausen, Germany). *P. falciparum* RNA was isolated from parasitized red blood cells using the RiboPure^TM^ RNA Purification kit, blood (Thermo Fisher Scientific, Oberhausen, Germany). The alternate protocol provided by the manufacturer for the isolation of small RNAs with subsequent DNase I treatment was used. Concentrations and absorbance were measured using a NanoVue^TM^ Plus spectrophotometer (GE Healthcare Life Sciences, Solingen, Germany). The integrity of the RNA was determined via Experion^TM^ (Bio-Rad Laboratories, Munich, Germany) analysis using RNA StdSens Chips, following the manufacturer's protocol. RNA used for experiments showed A_260_:A_280_ values of >2.0 and an RQI of >7. *Plasmodium* DNA was isolated with Trizol Reagent (Thermo Fisher Scientific, Oberhausen, Germany) and subjected to including RNAse A digestion (Thermo Fisher Scientific, Oberhausen, Germany). Concentration and absorbance were measured using a NanoVueTM Plus spectrophotometer (GE Healthcare Life Sciences, Solingen, Germany). DNA used for experiments showed an A_260_:A_280_ value of ~1.8.

### Stimulation and Treatment of Cells

Cells were stimulated in duplicates with TLR ligands, oligonucleotides, pathogens and pathogen-derived RNA as indicated. One hundred nanograms per milliliters LPS was used if not stated differently. Uninfected red blood cells (uRBC) or RBC infected with *P. falciparum* (iRBC) were added to cells as indicated. TLR7/8/9 ligands were complexed with polyL-Arginine, 5–15 kDa (p-Arg) as delivery agent. In brief, 360 ng p-Arg (Sigma-Aldrich, Schnelldorf, Germany) were added to 200 ng RNA or DNA for 10 min/ RT in 15 μl PBS and if not indicated differently a final concentration of 1 or 4 ug DNA or RNA/ ml was added to human immune cells and 10 ug DNA or RNA/ ml to THP-1 cells. Plasmid DNA (pBluescript) was used for dsDNA and transfected with Lipofectamine2000 (Thermo Fisher Scientific, Oberhausen, Germany) according to manufacturer's recommendations at a final concentration of 1 μg/ml. TLR7- and TLR8-expressing 293 cells were transfected with an NF-κB-gluc reporter plasmid and an EF1-Promoter-SEAP expression plasmid (as transfection control). Twelve hours after transfection, medium was changed and cells were stimulated with TLR ligands and reporter activity was measured after 16 h. To block endosomal acidification and TLR recognition, 10 μM chloroquine (Sigma-Aldrich, Schnelldorf, Germany) or 50 nM bafilomycin (Invivogen, Toulouse, France) were added 1 h before stimulation. To block NLRP3 and caspase-1 activation, CP-456,773/CRID3 (Sigma-Aldrich, Schnelldorf, Germany) and Z-YVAD-fmk (Enzo Life Sciences), respectively, were added 1 h before stimulation. IL-18 was blocked using IL-18BP (kindly provided by P. Bufler and Merck Serono) and IL-1β by IL-1 receptor antagonist (Anakinra, Swedish Orphan Biovitrum AB, Stockholm, Sweden). IL-12p70 was blocked using anti-IL-12p70 (R&D Systems, Wiesbaden-Nordenstadt, Germany). Recombinant IL-12p70 (ImmunoTools, Friesoythe, Germany) and IL-18 (MoBiTec, Göttingen, Germany) were used as controls.

### Cytokine Assays

After supernatant collection at the indicated time points, cytokines were measured in cell culture supernatants by the IFN-α module set (Bender MedSystems; Graz, Austria), human IFN-γ/ IL-1β/ TNF alpha or IL-12p70 ELISA set (all BD Pharmingen, Heidelberg, Germany), respectively. IL-18 was determined via alphaLISA from Perkin Elmer (Waltham, USA). All procedures were performed according to manufacturer's recommendations.

### Flow Cytometry Analysis

Intracellular staining was performed after incubation with brefeldin A (1 μg/ml, Sigma-Aldrich, Schnelldorf, Germany) for 4 h. Subsequently, the cells were surface stained and fixed of the cells with PBS/ 4% paraformaldehyde. Then cells were permeabilized with 0.5% saponin (Carl Roth, Karlsruhe, Germany) and intracellularly stained with IFN-γ-Alexa647 (BD Bioscience, Pharmingen, Heidelberg, Germany). To determine the expression of the activation marker CD69, the cells were harvested and stained in PBS containing 2% FCS (Thermo Fisher Scientific, Oberhausen, Germany) with CD69-PE-Cy7, CD3-Horizon V450, CD56-PE (BD Pharmingen, Heidelberg, Germany) for 15 min at 4°C in the dark, washed and analyzed via flow cytometry. Degranulation of NK cells was determined by measuring CD107a-PE, CD3-FITC, CD56-APC (BD Pharmingen, Heidelberg, Germany) and Vγ9-Alexa488 (for γδT cell staining (78) as generous gift from Dietrich Kabelitz and Daniela Wesch). Human PBMCs were co-cultured with tumor cells and CD107a-PE was added for 1 h. Degranulation was blocked with 5 μg/ml Monensin A (Sigma-Aldrich, Schnelldorf, Germany) for an additional 3 h. Flow cytometric analysis was performed using the LSR II from BD Bioscience and data were analyzed with FlowJo® (Tree Star, Switzerland).

### Statistics

Statistical analysis was performed using non-parametric tests. Wilcoxon matched-pairs signed rank test was used to compare paired groups. In case of multiple comparisons, Friedman test was used and corrected according to Bonferroni. ^*^indicates a *p* < 0.05; ^**^ < 0.01, and ^***^ < 0.001.

## Ethics Statement

Freshly prepared buffy coats and erythrocytes from human donors were obtained from the Institute for Experimental Hematology and Transfusion Medicine, University Hospital Bonn, Germany with the donors' written informed consent. Approval was given by the ethics committee of the University Hospital Bonn (167/11).

## Author Contributions

CC, BH, EB, TZ, JD-P, MN, and BP performed the experiments. CC, EB, TZ, MS, BK, AH, and GH conceived experiments. JFS, SS, and BS supported experiments with *P. falciparum* material. CC, EB, and GH wrote the manuscript. All authors revised the manuscript.

### Conflict of Interest Statement

The authors declare that the research was conducted in the absence of any commercial or financial relationships that could be construed as a potential conflict of interest. The reviewer MG and handling editor declared their shared affiliation at the time of the review.
