# Supplementary figures and images for: Human TLR8 Senses RNA From Plasmodium falciparum-Infected Red Blood Cells Which Is Uniquely Required for the IFN-γ Response in NK Cells
Source: Front Immunol. 2019 Mar 27;10:371. doi: 10.3389/fimmu.2019.00371 (PMC6445952; doi:10.3389/fimmu.2019.00371)

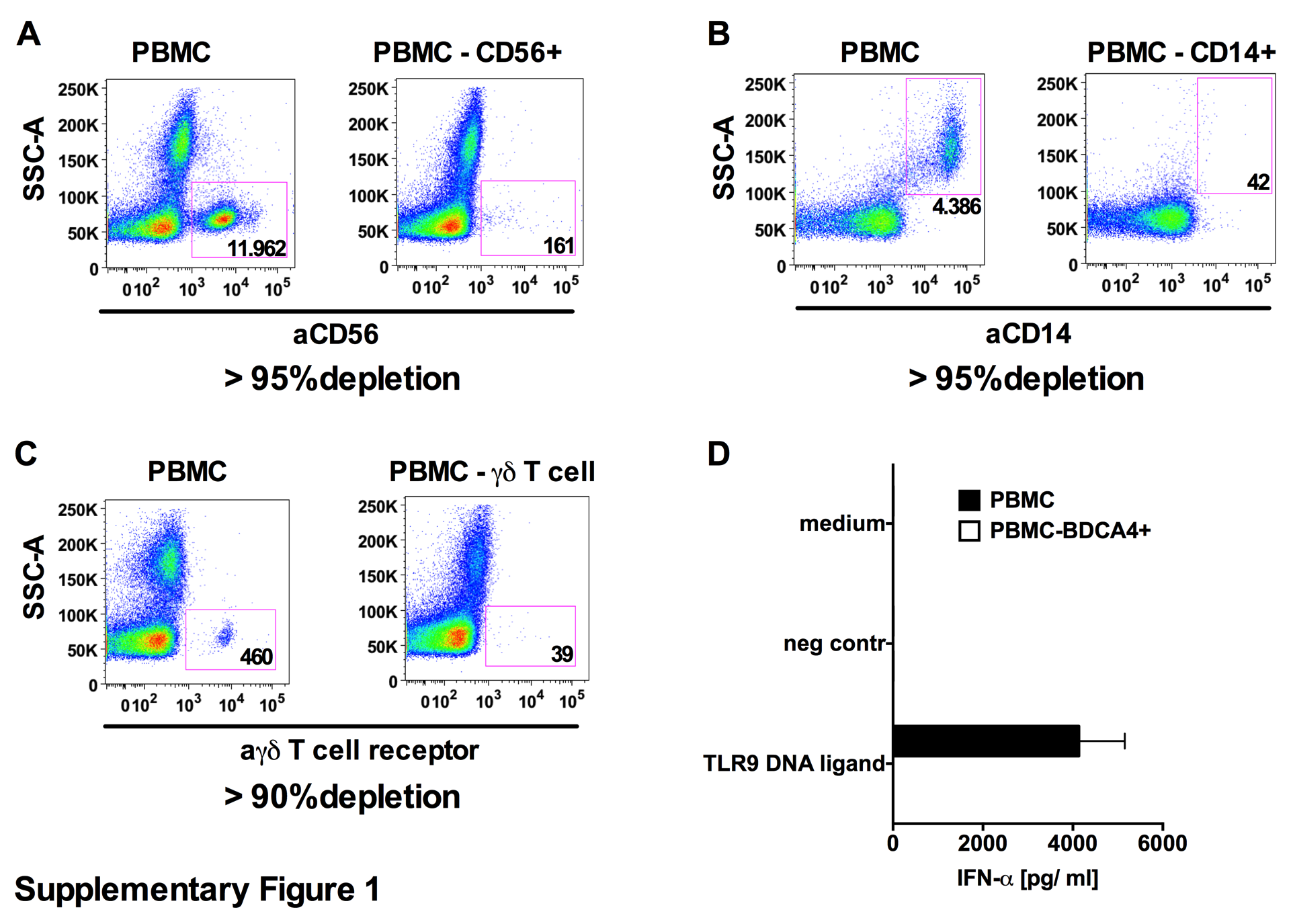

Supplement: Supplementary file 1 [file Image_1.TIFF]

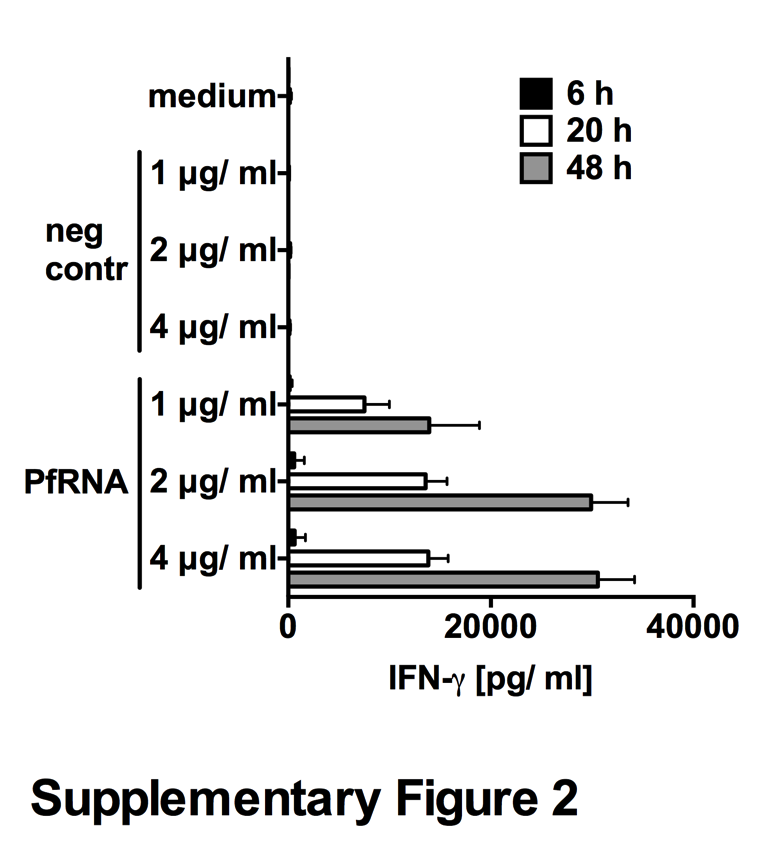

Supplement: Supplementary file 2 [file Image_2.TIFF]

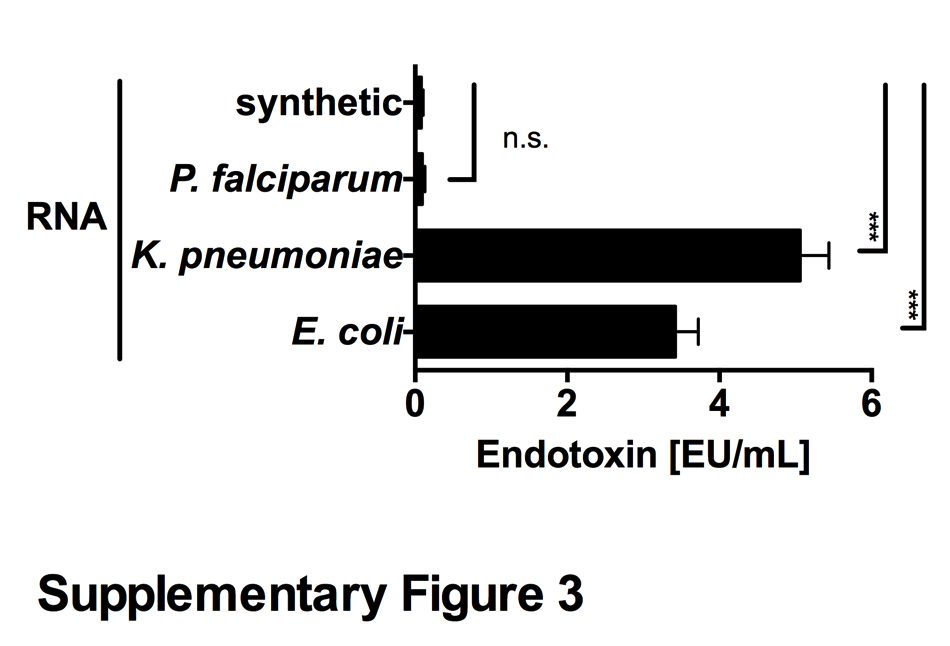

Supplement: Supplementary file 3 [file Image_3.TIFF]

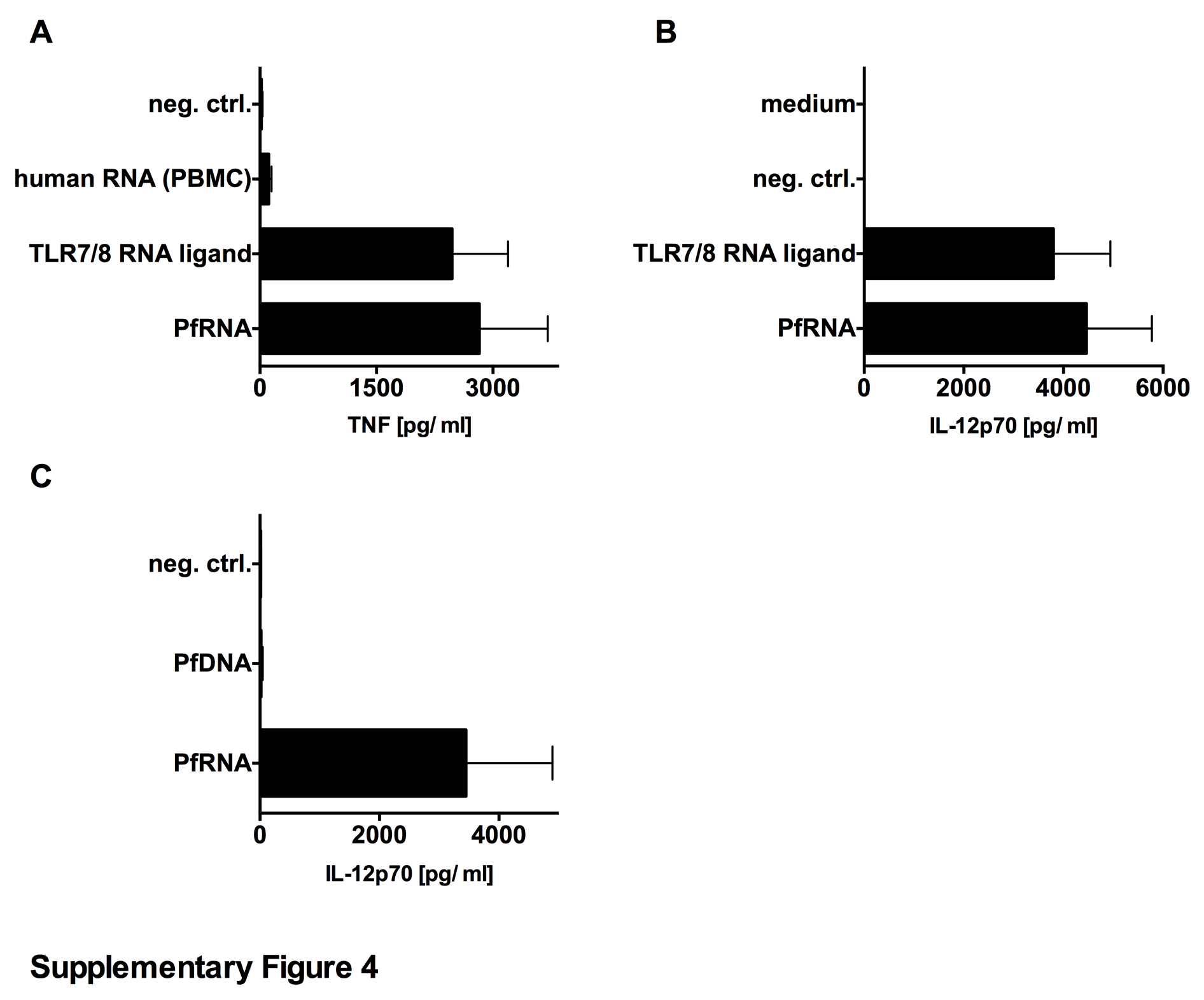

Supplement: Supplementary file 4 [file Image_4.TIFF]

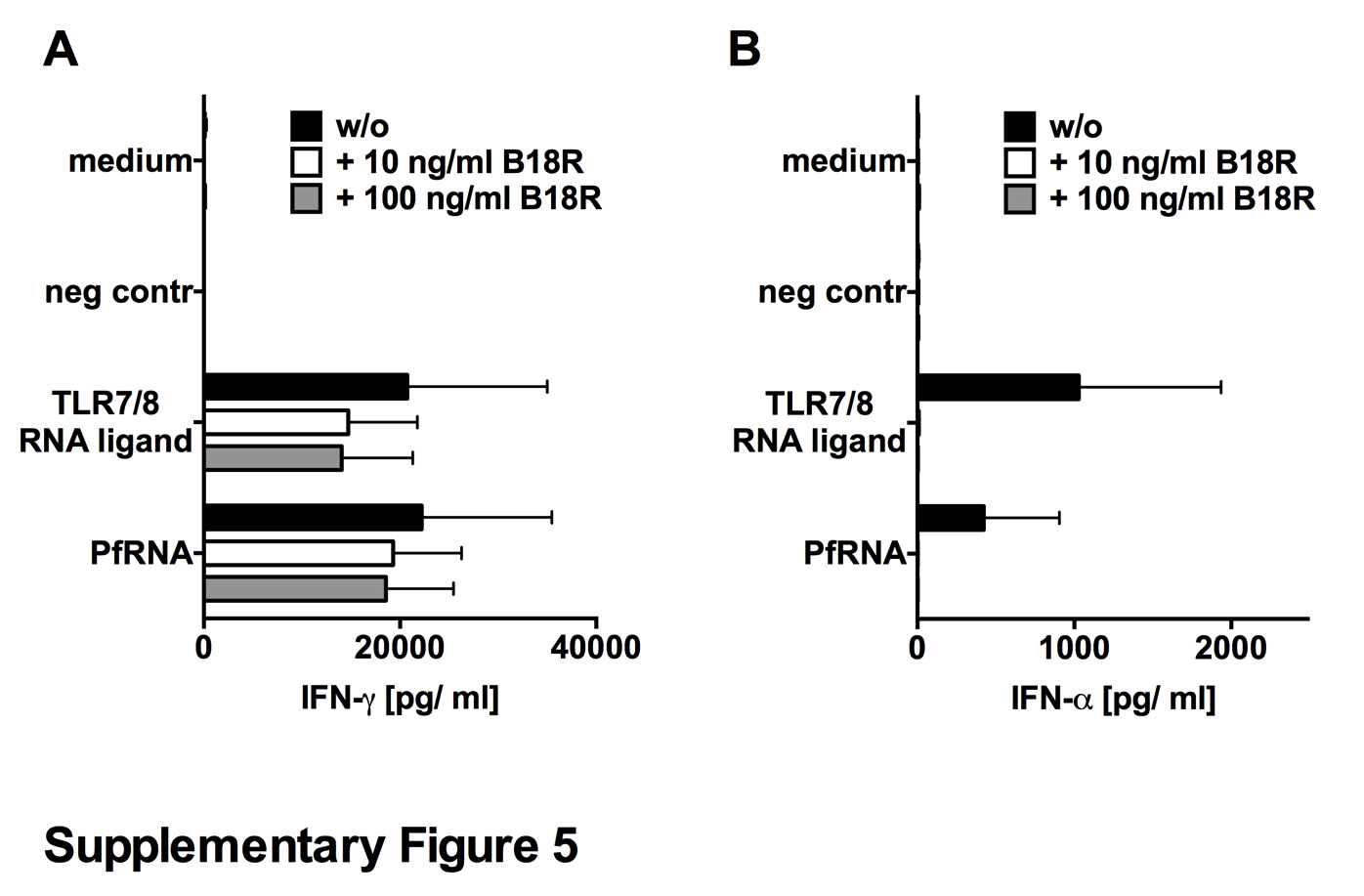

Supplement: Supplementary file 5 [file Image_5.TIFF]

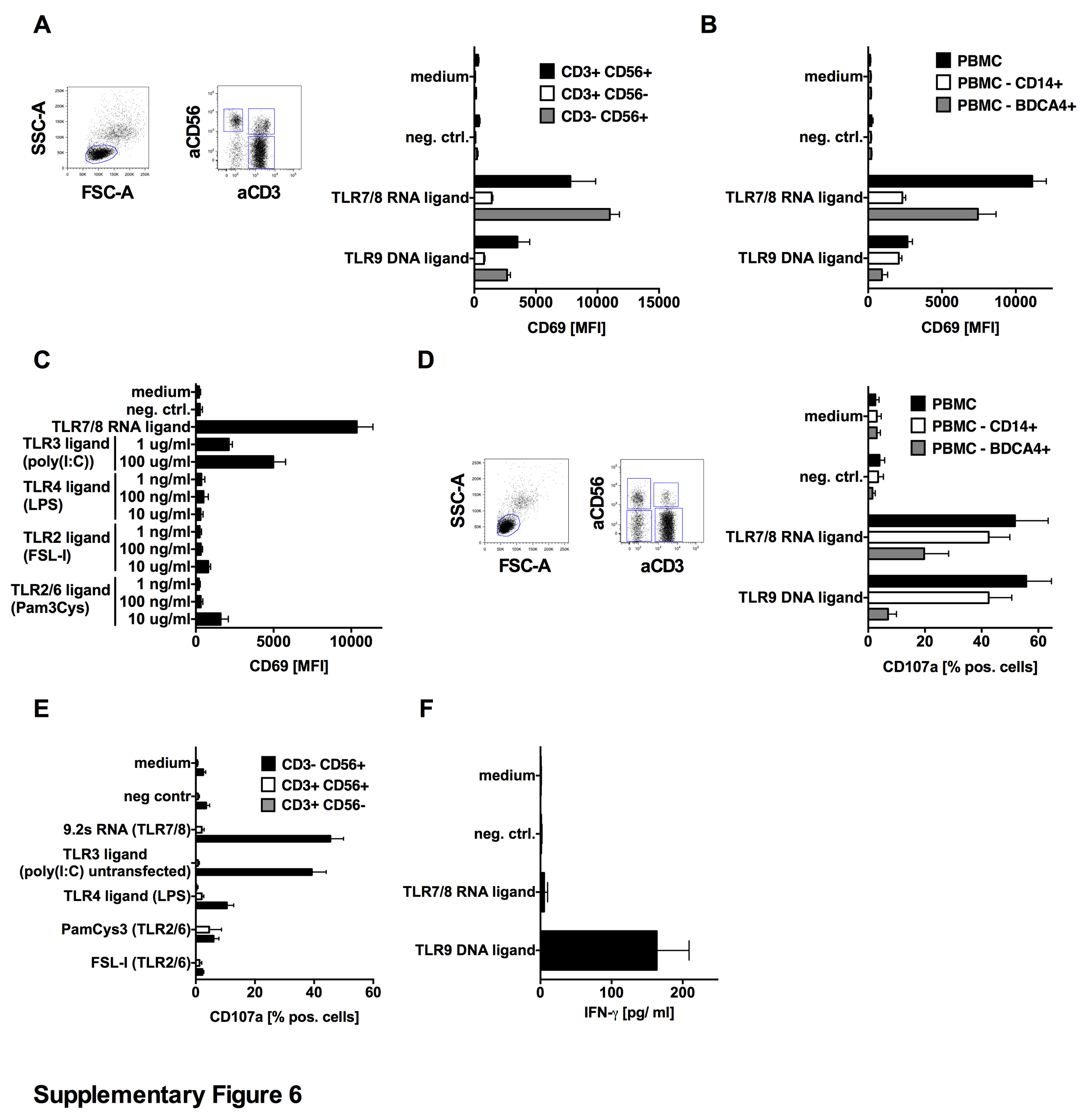

Supplement: Supplementary file 6 [file Image_6.TIFF]
