# Supplementary material for: Human TLR8 Senses RNA From Plasmodium falciparum-Infected Red Blood Cells Which Is Uniquely Required for the IFN-γ Response in NK Cells
Source: Front Immunol. 2019 Mar 27;10:371. doi: 10.3389/fimmu.2019.00371 (PMC6445952; doi:10.3389/fimmu.2019.00371)
Supplement: Supplementary file 7 [file Data_Sheet_1.PDF]

Supplementary Information

Materials and Methods

Isolation and culture of mouse splenocytes

Mouse spleens were digested for 30min with 0.5mg/ml collagenase, 100µg/ml DNAse I (all Sigma-Aldrich, St. Louis, MO), 20mM HEPES (Carl Roth, Karlsruhe, Germany), 0.1% β-mercaptoethanol (Gibco, Darmstadt, Germany) at 37°C with intermediate mashing. Digestion was stopped using RPMI1640 (Biochrom, Berlin, Germany) + 10% (v/v) FCS (Invitrogen, Karlsruhe, Germany), lumps were removed using gauze (41µm, Bückmann, Mönchengladbach, Germany), RBC were lysed using BD Pharm Lyse (BD Pharmingen). 5x105 mouse cells were cultured in RPMI 1640 (Biochrom, Berlin, Germany) 10% (v/v) FCS (Invitrogen, Karlsruhe, Germany), 1 mM L-Glutamine, 100U/ml penicillin and 100µg/ml streptomycin (Sigma-Aldrich, St. Louis, MO) in 96-well plates.

Endotoxin quantification

Endotoxin was quantified using the Pierce™ Chromogenic Endotoxin Quant Kit (Thermo Fisher Scientific), which uses amoebocyte lysate (LAL) in combination with a chromogenic substrate. The kit was used in accordance with the manufacturer's instructions and has a detection limit of 0.1 EU/mL endotoxin.

Figure legends

Supplementary Figure 1:

Human PBMC were depleted of CD56+ cells (A), CD14+ cells (B) or cells expressing the $\gamma\delta$ T cell receptor (C). Purity of depletion is depicted for representative donors.

(E) Human PBMC with or without depleted CD304+ cells were stimulated with the TLR9 ligand CpG 2216 or poly$(CA)_{10}$ or medium as negative controls. After 24h IFN-α was measured in the supernatant. Graph shows mean of n=4 donors +/- SEM.

Supplementary Figure 2:

Human PBMC were stimulated with PfRNA or poly$(CA)_{10}$ as negative control in the indicated concentrations. After indicated incubation time IFN-γ was measured in the supernatant. Graph shows mean of n=3 donors +/- SEM.

Supplementary Figure 3:

Endotoxin quantification was performed using an end-point chromogenic detection assay. A final concentration of 10µg/mL total RNA from the gram-negative bacteria *Klebsiella pneumoniae* (*K. pneumoniae*) *and Escherichia coli* (*E. coli*) as well as *Plasmodium falciparum* (*P. falciparum*) was subjected to quantification. Synthetic RNA was used as a negative control. Endotoxin concentration was calculated via linear regression of a standardized LPS titration. These data are representative of 2 independent experiments.

Supplementary Figure 4:

(A-C) Human PBMC (A, C) or human isolated monocytes (B) were stimulated with a TLR7/8 ligand (9.2s RNA), *P. falciparum*-RNA (PfRNA), *P. falciparum*-DNA (PfDNA), human RNA from PBMC or an inert RNA (neg. ctrl.) as indicated. After 24h, TNF (A) or IL-12p70 (B, C) was analyzed in the cellular supernatant. The data shown are mean ±SEM of 4 compiled donors.

Supplementary Figure 5:

(A+B) Human PBMC were stimulated with PfRNA, a TLR7/8 RNA ligand or negative controls (medium only or poly$(CA)_{10}$) and additionally treated with B18R to inhibit type

I IFN activity in the supernatant. After 24h, the supernatant was analyzed for IFN-γ and IFN-α by ELISA. Graph shows mean +/- SEM of n=4 donors.

Supplementary Figure 6:

(A+B) Human PBMC with or w/o depletion of $CD14^{+}$ monocytes or BDCA4+ pDC were stimulated with a TLR9 ligand (CpG 2006) or a TLR7/8 ligand (9.2s RNA) and CD69 expression on indicated cell types (A) or CD3 neg CD56 pos cells (B) were analyzed by FACS. Mean fluorescence intensity (MFI) is depicted. Graphs show mean ± SEM of 5-6 donors. (C) Done as described in (B) but ligands for TLR2/3/4/6 were used. Graph shows mean ±SEM of 2 donors. (D+E) Done as described in (A) but PBMC were treated with additional TLR ligands as indicated and translation was blocked with Brefeldin A after 12h, incubated with tumor cells (A549) and percentage of CD107a expressing cells was analyzed by FACS. Graph shows mean ± SEM of 4 donors. (F) Spleen cells from mouse were stimulated with a TLR7/8 ligand (9.2s RNA) or TLR9 ligand (CpG 2006) or with an inert RNA (neg. ctrl.). After 24h, supernatant was analyzed for IFN-γ by ELISA. Graph shows mean ± SD of 2 experiments.

Supplementary Table 1:

Table shows sequencing results of CRISPR/CAS generated InDel of THP-1 cell lines
