# Supplementary material for: Human TLR8 Senses RNA From Plasmodium falciparum-Infected Red Blood Cells Which Is Uniquely Required for the IFN-γ Response in NK Cells
Source: Front Immunol. 2019 Mar 27;10:371. doi: 10.3389/fimmu.2019.00371 (PMC6445952; doi:10.3389/fimmu.2019.00371)
Supplement: Supplementary file 8 [file Table_1.pdf]

	InDel TLR7	Sequence*	InDel TLR8	Sequence*
**THP-1 WT**	-	CACCATTA/ACCACATAC		AACTTCCTGTA/GTCGACGATT
**TLR7 #1**	-1 bp	TCACCATTA.CCACAT	-	
**TLR7 #2**	+1 bp	CACCATTA[+A]ACCACATAC	-	
**TLR8 #1**	-		-23 bp	TTATTG...CAGGAAGTTC
**TLR8 #2**	-		-1 bp	TCGTCGAC.ACAGGAAG
**TLR7/8 #1**	+1 bp	CACCATTA[+A]ACCACATAC	+1 bp	AATCGTCGAC[+A]TACAGGAA
**TLR7/8 #2**	+1 bp	CACCATTA[+A]ACCACATAC	5 bp	CAGCAATC...CTACAGGAA
* THP-1 are derived from a male patient and therefore carry only one X chromosome/ indicates Cas9 cut site				

Supplementary Table 1
